# Expression of the cellular prion protein by mast cells in white-tailed deer carotid body, cervical lymph nodes and ganglia

**DOI:** 10.1080/19336896.2024.2402225

**Published:** 2024-09-16

**Authors:** Anthony E. Kincaid, Nathaniel D. Denkers, Erin E. McNulty, Caitlyn N. Kraft, Jason C. Bartz, Candace K. Mathiason

**Affiliations:** a Department of Pharmacy Sciences, School of Pharmacy and Health Professions, Creighton University, Omaha, NE, USA; b Department of Medical Microbiology and Immunology, School of Medicine, Creighton University, Omaha, NE, USA; c Department of Microbiology, Immunology and Pathology, College of Veterinary Medicine and Biomedical Sciences, Colorado State University, Fort Collins, CO, USA

**Keywords:** Carotid body, chronic wasting disease, lymph node, mast cell, nodose ganglion, prions, superior cervical ganglion, white-tailed deer

## Abstract

Chronic wasting disease (CWD) is a transmissible and fatal prion disease that affects cervids. While both oral and nasal routes of exposure to prions cause disease, the spatial and temporal details of how prions enter the central nervous system (CNS) are unknown. Carotid bodies (CBs) are structures that are exposed to blood-borne prions and are densely innervated by nerves that are directly connected to brainstem nuclei, known to be early sites of prion neuroinvasion. All CBs examined contained mast cells expressing the prion protein which is consistent with these cells playing a role in neuroinvasion following prionemia.

## Introduction

Chronic wasting disease (CWD) is an emerging fatal prion disease affecting deer, elk, and moose that has been spreading in North America since it was first identified in captive deer in Colorado in the 1960s [1]. As of March 2024, CWD has been identified in 32 states: four Canadian provinces, Finland, Norway, Sweden, and South Korea [2]. The spread of CWD has been facilitated by efficient horizontal and vertical transmission mechanisms in captive and wild animal populations [3–6]. Horizontal transmission of CWD is likely to occur via direct and indirect exposure to infectious prions that have been identified in bodily fluids and tissues, such as blood, urine, faeces, saliva, decaying carcasses, and antler velvet [7–11]. Both oral and nasal routes of exposure to infectious prions have been demonstrated to cause CWD [12–21]. Following either route of inoculation, there is a subsequent and persistent prionemia that results in a systemic spread of infectious prions [22,23].

Mammalian carotid bodies (CBs) are small specialized sensory structures located bilaterally near the bifurcation of the common carotid arteries in the neck. They are surrounded by a connective tissue capsule with septae that project into the middle of the organ, dividing it into compartments containing collections of cells [24]. Some of these cells are sensory neurons that detect levels of oxygen and carbon dioxide in the blood and play a role in the rapid adjustment of the respiratory rate to meet the functional demands of the animal [24–26]. There is a dense blood supply to CBs, with a large concentration of fenestrated capillaries and a high blood flow rate, considered to be the highest for any tissue in the body [27–29]. Thus, chemosensitive cells of CBs, referred to as type II cells, are continually exposed to the contents of the blood [reviewed in 24]. CBs are innervated by peripheral branches of the carotid sinus nerve, a branch of the glossopharyngeal nerve, and to a lesser extent by efferent branches of sympathetic postganglionic neurons [30–32]. Relevant to prion pathogenesis is the fact that central processes of the carotid sinus nerve terminate in the nucleus of the solitary tract (NTS) in the medulla [33–35] and that sympathetic postganglionic neurons are synaptically linked to cells in the intermediolateral cell column [IML] of the thoracic spinal cord. The pathogenic isoform of the prion protein has been shown to accumulate in the NTS and IML following oral inoculation [13,36].

Given their significant exposure to blood and their direct connection to central nervous system (CNS) areas involved in prion neuroinvasion, we sought to determine whether cells in white-tailed deer [WTD] CB expressed the normal isoform of the cellular prion protein (PrP^C^) or were sites of deposition of the disease-associated isoform of the prion protein (PrP^CWD^) in infected animals using immunohistochemistry (IHC).

## Results

**Figure 1.**
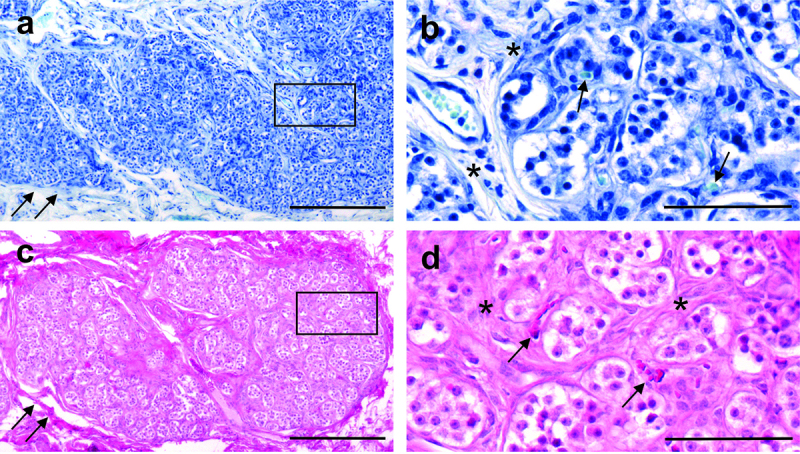
Carotid bodies (CB) from white-tailed deer (WTD; animal #1450) can be identified by staining tissue sections with either toluidine blue (TB; panels a, b) or haematoxylin and eosin (H&E; panels c, d). Low power views (panels a, c) show that CBs are irregular-shaped lobulated structures surrounded by connective tissue capsules (indicated by arrows; more obvious in H&E sections). Bands of connective tissue extend towards the middle of the CBs separating the structure into clusters of cells. Medium power views (panels b, d) show the connective tissue bands separating the clusters of cells (indicated by asterisks). Note the presence of numerous blood vessels, some of which contain red blood cells (indicated by arrows). Scale bars: panels a, c = 200 µm; panels b, d = 50 µm.

CBs (*n* = 19) were collected from 11 WTD who were inoculated via oral or nasal routes with prion-infected or uninfected tissues. The sex, genotype, and inoculation details of the animals are listed in Table 1. Tissues harvested from these animals have been used in a previous study and details about the QuIC and IHC methods and analysis are as reported [18]. CBs were identified using light microscopy after sectioning the tissue blocks on a microtome and staining the sections using haematoxylin and eosin (H and E) or toluidine blue (TB). WTD CBs have a characteristic appearance that is easily identified within the loose connective tissue sheath surrounding the carotid blood vessels (Figure 1) and are similar in size and morphology to human CBs [37].

**Table 1.** Inoculum, route of inoculation, incubation period, age and presence of PrP^Sc^ at termination of white-tailed deer used in this study*.

Animal	Genotype	Sex	Inocula Source/dose	Inoculation Regimen	Route of inoculation	MPI*	Age at death	Terminal assays
1 (1306)	GS	F	(+) saliva/300 ng	3×100 ng over 3 weeks	oral	47	58 months	QuIC (+) IHC (+)
2 (1438)	GG	F	(+) saliva/300 ng	1×300 ng Single bolus	oral	39	49 months	QuIC (+) IHC (+)
3 (1436)	GG	M	(+) saliva/300 ng	10×30 ng Over 12 weeks	oral	28	37 months	QuIC (-) IHC (-)
4 (1440)	GG	M	(+) saliva/300 ng	10×30 ng Over 12 weeks	oral	28	37 months	QuIC (-) IHC (-)
5 (1442)	GG	M	(+) saliva/300 ng	10×30 ng Over 12 weeks	oral	28	37 months	QuIC (-) IHC (-)
6 (1450)	GG	F	(+) brain/300 ng	10×30 ng Over 12 weeks	oral	28	37 months	QuIC (-) IHC (-)
7 (1841)	GG	F	(-) brain/RPLN 0.5 mg each	1×0.5 mg Single bolus	intranasal	3	14 months	QuIC (-) IHC (-)
8 (1844)	GG	M	(+) brain/0.5 mg	1×0.5 mg Single bolus	intranasal	3	14 months	QuIC (+) IHC (-)
9 (1846)	GG	M	(+) brain/0.5 mg	1×0.5 mg Single bolus	intranasal	3	14 months	QuIC (+) IHC (+)
10 (1960)	GG	M	(+) RPLN/0.5 mg	1×0.5 mg Single bolus	intranasal	3	13 months	QuIC (+) IHC (+)
11 (1972)	GS	F	(+) RPLN/0.5 mg	1×0.5 mg Single bolus	intranasal	3	13 months	QuIC (+) IHC (-)

*QuIC and IHC methods and analysis are detailed in reference [18].

**MPI = Months Post Inoculation.

RPLN = retropharyngeal lymph node.

**Figure 2.**
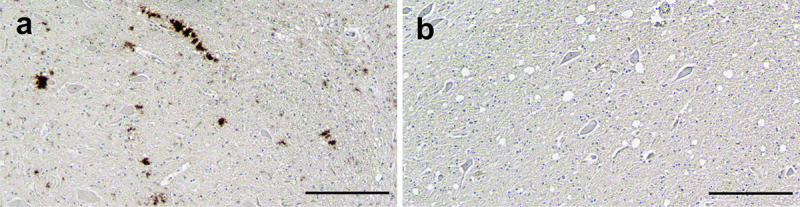
The 8H4 antibody was used to demonstrate the presence of the prion protein in WTD tissue. Tissue sections collected from the obex of an infected WTD (panel a) shows the presence of the pathogenic isoform of the prion protein (PrP^CWD^) in a clinically-ill deer (animal #817). The area inside the box is enlarged in the inset. The plaque-like immunoreactivity was only noted in the obex of infected animals. Tissue sections taken from the obex of an uninfected deer (animal #955) and processed concurrently (panel b) show a lack of PrP^CWD^ immunoreactivity. Scale bars: panels a, b = 200 µm.

**Figure 3.**
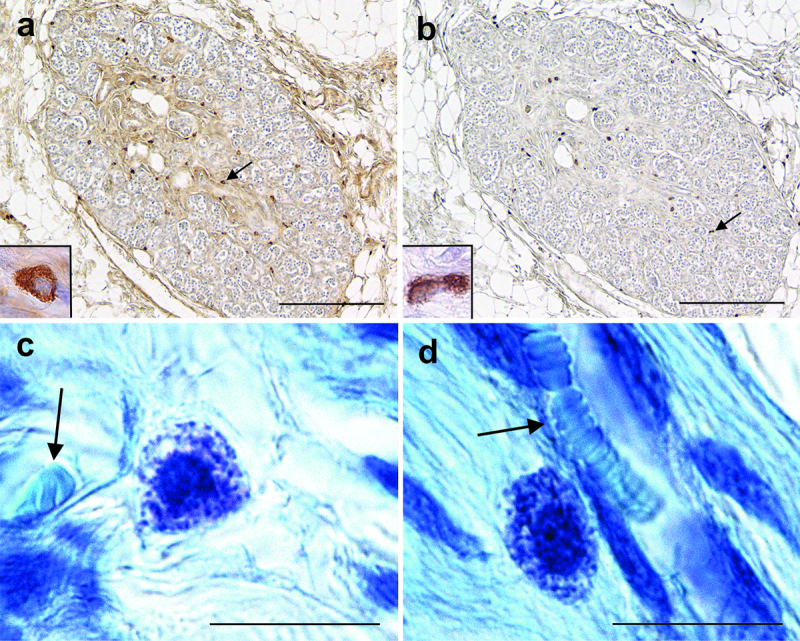
Mast cells in the WTD CB express the prion protein. CBs from an animal (animal # 1436) were identified using the prion antibody, 8H4, (panel a) or a mast cell specific antibody (panel b). Note that the distribution, size and morphology of the cells is the same in the two panels. Arrows indicate a single cell enlarged in the insets of panels a and b. Note the presence of large granules in the cells, a characteristic feature of mast cells, which were immunoreactive using either antibody. Replacement of the primary antibodies with the appropriate isotype control resulted in the complete absence of any PrP^C^ immunoreactivity (Figure 3c) or mast cell immunoreactivity (Figure 3d). Mast cells can also be identified by staining with TB/AS (panel e). To confirm that mast cells express PrP^C^ tissue sections were IHC processed using the 8H4 antibody followed by counterstaining with TB/AS. Only one population of cells was stained and was immunoreactive for 8H4 and contained the granules characteristic of mast cells (panel f; animal # 1438). Arrows indicate stacks of red blood cells in capillaries adjacent to the mast cells, demonstrating the proximity of mast cells to blood in the CBs (panels e, f). Scale bars: panels a-b = 200 µm. Panels c-d = 100 µm. Panels e, f = 10 µm.

The 8H4 antibody, which recognizes an epitope between residues 145–180 of the human prion protein, was used to identify the prion protein in WTD tissue blocks using IHC [38]. To determine the efficacy of 8H4 in formalin-fixed paraffin-embedded WTD tissue, we examined positive and negative control tissue sections containing the obex from deer that were established to be either infected or uninfected. There was scattered plaque-like staining across a wide area of the obex in an infected and clinically ill animal. The PrP^CWD^ was characterized by dense, plaque-like staining of extracellular aggregates of immunoreactive elements with undefined contours (Figure 2a inset). There was a complete absence of immunoreactive staining in the obex of an uninfected/healthy animal, showing that the antibody recognizes PrP^CWD^ (Figure 2b). IHC processing of tissue sections containing CBs from each of the 11 deer used in this study using the 8H4 antibody resulted in the staining of a population of cells scattered throughout the CB that were notable for the presence of large granules in the cytoplasm (Figure 3a). These cells were observed to have a similar distribution, size, and morphology as mast cells that were identified in the same animals using a mast cell-specific antibody (Figure 3b). Replacement of the primary antibodies with appropriate isotype controls resulted in a lack of staining in the tissue sections indicating the specificity of the antibodies (Figure 3c,d). To determine whether the cells identified using the two methods were the same population of cells in WTD, tissue sections were stained with TB/ammonium sulphate (TB/AS) to identify mast cells (Figure 3e) or were IHC processed using the 8H4 antibody and then counterstained with TB/AS. Following the combination of 8H4 IHC and TB/AS counterstaining, a single population of cells that were both TB-positive and immunoreactive for PrP^C^ was identified (Figure 3f), indicating that mast cells express PrP^C^. Prion protein-expressing mast cells were often located in proximity to capillaries, where they could be exposed to blood-borne infectious prions (Figure 3e, f). PrP^CWD^ deposition was not detected in any of the WTD CBs, including CBs collected from animals that were determined to be infected by IHC or real-time quaking-induced (RT QuIC) assays (Table 1). The 8H4 antibody recognized PrP^C^ in mast cells in the CBs of all animals, infected and uninfected; no PrP^CWD^ was present in the CBs of any of the animals.

**Figure 4.**
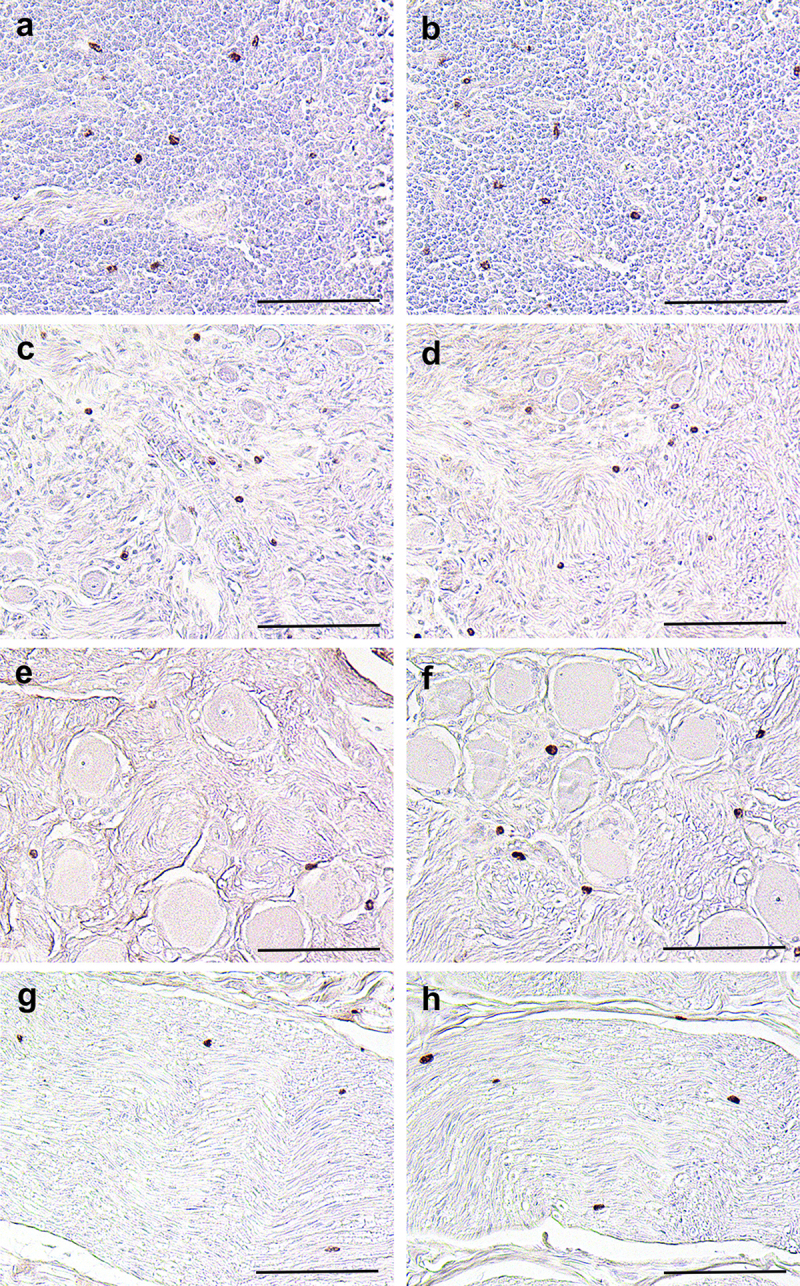
Mast cells in WTD structures located near the CBs also express the prion protein. Tissue sections containing cervical lymph nodes (panels a, b; animal #1442), superior cervical ganglia (panels c, d; animal #1306), nodose ganglia (panels e, f; animal #1438) and nerve (panels g, h; animal #1438) were stained using either 8H4 antibody (panels a, c, e, g) or mast cell antibody (panels b, d, f, h). Note the number, distribution, size and morphology of the cells is the same in tissue sections processed using either antibody and that there was no difference between tissues collected from infected or uninfected tissues. Scale bars: panels a-h = 100 µm.

Some of the CB tissue blocks collected from the WTD also contained lymph nodes, superior cervical ganglia, nodose ganglia (inferior ganglia of the vagus nerve), and associated nerves. Cells that were immunoreactive using the 8H4 and mast cell antibodies were identified in these structures (Figure 4a-h). Similar to the CBs, the cells that were immunoreactive for the two antibodies were of comparable size, morphology, distribution and were filled with granules and were determined to be the same population of cells. Thus, mast cells expressing the prion protein were present in cervical lymph nodes, sympathetic ganglia, vagal afferent ganglia, and peripheral nerves. There was no PrP^CWD^ detected in any of the lymph nodes, ganglia or nerves of these animals.

## Discussion

The 8H4 antibody recognizes the full-length glycoforms as well as the truncated forms of PrP^C^ in several primate species, cow, sheep, and squirrel [38]. Moreover, 8H4 also immunostained PrP^CWD^ in murine and human tissues collected from infected subjects, indicating that this antibody can recognize both normal and disease-associated isoforms of the prion protein [38]. This study provides evidence that the 8H4 antibody can detect both PrP^C^ and PrP^CWD^ in paraffin-embedded WTD tissues. PrP^C^ was expressed by mast cells in the CB of every animal examined, regardless of inoculation status. While a thorough quantitative assessment of mast cell numbers was not performed, there were no apparent differences in the distribution or density of prion protein-expressing mast cells in infected (positive by both RT-QuIC and IHC assays) versus uninfected animals (negative by both RT-QuIC and IHC assays; Table 1).

**Figure 5.**
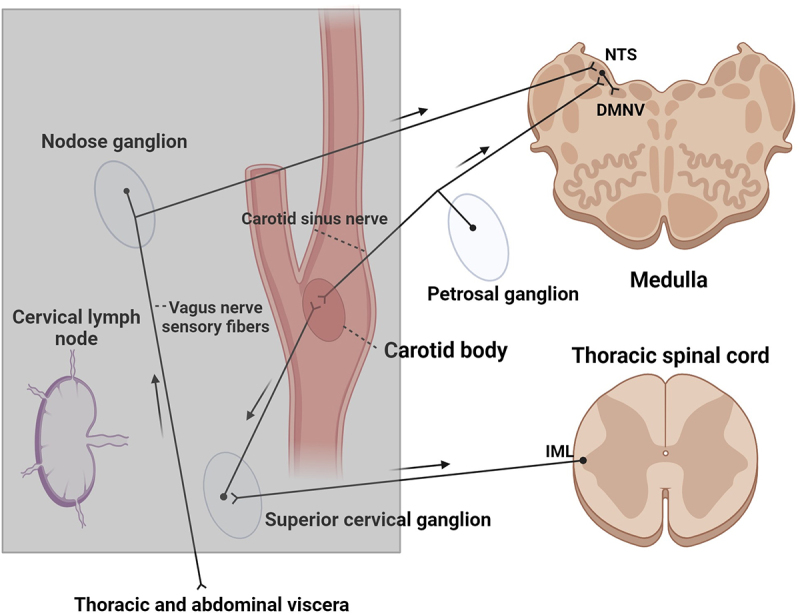
Schematic representation of potential routes of neuroinvasion following CB exposure to prions in blood. Black circles represent neuronal cell bodies, lines represent axons, and the ‘v’ line split represents axon terminals. Structures shown within grey shaded area of the figure were located within tissue blocks taken from WTD exposed to prion-infected tissues as listed in Table 1 (not all structures were in all tissue blocks). Two potential routes of prion neuroinvasion from CBs are synaptically linked to early sites of prion neuroinvasion: the nucleus of the solitary tract (NTS) and the intermediolateral cell column of the thoracic spinal cord (IML). The first pathway is via branches of the carotid sinus nerve that terminates in the NTS in the brainstem. The sensory cell bodies of these axons are located in the petrosal ganglion. The NTS is synaptically linked to the dorsal motor nucleus of the vagus (DMNV). The second pathway is via the sympathetic postganglionic fibres that innervate the CBs; the cell bodies of these fibres are located in the superior cervical ganglion (SCG). These neurons are synaptically linked to sympathetic preganglionic fibres that originate from sympathetic preganglionic neurons in the IML of the thoracic spinal cord. The arrows indicate the direction of transport of prions into the CNS. Tissue blocks also included the nodose ganglion (NG) which is the location of vagal sensory neurons that innervate thoracic and abdominal viscera, including the Peyer’s patches of the ileum, known to contain PrP^CWD^ following oral exposure [13]; the fibres of these cells terminate in the NTS. Some of the sections included cervical lymph nodes (CLNs), known sites for prion replication [40]. The CBs, SCG, the NG, the nerves and CLNs all contained mast cells that expressed PrP^C^. This figure was created using BioRender.com.

Mast cells have been reported to express the prion protein in a human cell line [39], and more recently, mast cells in human CB were found to express PrP^C^ [37]. The identification of mast cells expressing PrP^C^ in the CBs of WTD reported here extends these findings to a species that is particularly susceptible to CWD transmission. Moreover, mast cells expressing PrP^C^ were noted in other tissues known to be involved in prion pathogenesis. PrP^C^-expressing mast cells were identified in the lymph nodes, sympathetic ganglia, vagal sensory ganglia, and nerves located close to the CBs in each tissue block containing these structures. The presence of mast cells expressing PrP^C^ in tissues known to replicate PrP^CWD^ (lymph nodes), and to be synaptically linked to CNS areas known to be involved in prion neuroinvasion (sympathetic ganglia, vagal afferent ganglia and nerves) suggests these cells may be involved in prion pathogenesis [12,13,36,40]. Mast cells release PrP^C^ when activated, and the list of bioactive substances that cause mast cell degranulation includes IgE antibodies, complement proteins, reactive oxygen species, neuropeptide neurotransmitters, and neurotrophic factors [reviewed in [41–43]]. Prion neuroinvasion could be facilitated by either the transport of blood-borne PrP^CWD^ by the carotid sinus and/or sympathetic nerves, or by the degranulation of mast cells in the CBs with the release of PrP^C^ which is converted to the pathogenic form of the prion protein and then transported by the same nerves (Figure 5). It is also possible that neuroinvasion could be facilitated by direct interactions between mast cells and nerves and/or by the direct migration of mast cells into the CNS, as has been reported [44–47]. The results reported here strengthen the hypothesis that mast cells may play a key role in the pathogenesis of prion diseases [48].

## Significance

To our knowledge, this is the first published histological description of CBs collected from deer. The location, morphology, and organization of WTD CBs are similar to descriptions of the CB anatomy of other species in that they are surrounded by connective tissue capsules with septae that divide them into lobules of cell clusters that are innervated by sensory and autonomic fibres and are densely vascularized [24,49,50]. The finding that mast cells expressing PrP^C^ are located in CBs, cervical lymph nodes, sympathetic ganglia, vagal afferent ganglia, and adjacent nerves of a natural host species for CWD is relevant to prion pathogenesis in that PrP^C^ is required for the spread and replication of the pathogenic isoform of the prion protein and each of these structures has been reported to replicate prions or is synaptically linked to areas of the CNS that accumulate PrP^CWD^ early in the neuroinvasive period of the disease [13,51–53].

## Materials and methods

Animals: Four (4) groups of white-tailed deer fawns were acquired through a long-standing collaboration with the University of Georgia Warnell School of Forestry and transported to the Colorado State University (CSU) indoor CWD research facility at 5 months of age. The ARRIVE guidelines were followed throughout the course of these studies [54]. All studies were approved by and conducted under strict guidelines of the CSU Institutional Animal Care and Use Committee (protocols 18-8396A; 18-7969A; 1476; 3575). Tissues from six of these animals were previously used in a published study [18], and the animals were identified by cohort number. Inocula were prepared to specific concentrations in 1X phosphate-buffered saline from either CWD-positive pooled saliva material, brain, retropharyngeal lymph node, or CWD-negative brain and administered as: 1.) 300ng positive saliva *Per Os* (PO) as 3-100ng doses over 3 weeks (*n* = 1; cohort 4 in reference 18); 2.) 300ng positive saliva PO as a single bolus dose (*n* = 1; cohort 6 in reference 18); 3.) 300ng positive saliva PO as 10-30ng doses over 12 weeks (*n* = 3; cohort 5 in reference 18); 4.) 300ng positive brain PO as 10-30ng doses over 12 weeks (*n* = 1; cohort 3 in reference 18); 5.) 0.5 mg negative brain and retropharyngeal lymph node atomized intranasally as a single bolus dose (*n* = 1); 6.) 0.5 mg positive brain atomized intranasally as a single bolus (*n* = 2); 7.) 0.5 mg positive retropharyngeal lymph node atomized intranasally as a single bolus (*n* = 2).

### Tissue collection

Blocks of tissue containing CBs were obtained during terminal tissue collection from 11 WTD. Carotid sheaths were identified bilaterally and isolated from the surrounding structures. Within the carotid sheaths, the bifurcation of the common carotid artery was identified, and a block of tissue approximately 2 cm wide by 2 cm long and 1 cm deep was excised. The tissue blocks were placed in tissue cassettes and immersion-fixed prior to embedding in paraffin.

### Histology and histochemistry

The histological and immunohistochemical methods used in this study have been previously described [37]. Tissue sections were cut at 7 µm on a rotary microtome and collected on glass slides. Briefly, tissue sections containing CBs were identified by staining with TB (Figure 1a,b), H and E (Figure 1c,d). Nineteen CBs were successfully obtained from 11 WTD (Table 1). A solution of 2% TB (Fisher; T161–25) and 5% ammonium sulphate (J.T. Baker; JT4628–01) was used to identify the mast cells. The metachromatic reaction selectively stained mast cell granules purple, which were discernible from the blue-stained nuclei of the surrounding cells (Figure 3e, f).

### Immunohistochemistry

Tissue sections were deparaffinized and treated with 10% formic acid (10 minutes) to facilitate antigen retrieval. Endogenous peroxidase was blocked using 3% hydrogen peroxide in methanol (20 minutes) and non-specific binding was blocked using 10% normal serum in 0.05% Tween in Tris-buffered saline (TTBS; 30 minutes). Following three rinses in TTBS tissue sections were incubated in a mouse monoclonal 8H4 prion antibody (Abcam, #61409) or mouse monoclonal anti-mast cell tryptase (DAKO. M7052) at 1:500 in TTBS with 0.3% normal horse serum at 35° F for 24 hours. Following three rinses in TTBS, the sections were incubated in biotinylated horse anti-mouse antibody at 1:500 (Vector, BA #2001) in TTBS with 0.3% normal horse serum for 1 hour at room temperature, then placed in avidin-biotin solution (1:200; Vector Laboratories, Burlingame, CA) in TTBS for 20–30 minutes at room temperature, and then reacted in filtered 0.05% diaminobenzidine tetrachloride (Sigma, St. Louis, MO) with 0.0015% H_2_O_2_ for 10–20 minutes. The sections were rinsed and counterstained with either haematoxylin or TB/ammonium sulphate, dehydrated using alcohol, cleared in xylene, and coverslipped using Cytoseal-XYL (Richard Allan Scientific, Kalamazoo, MI). Some tissue sections were processed in an identical manner, but with either the primary or secondary antibodies omitted, or with the same concentration of isotype control in place of the primary antibody (8H4: IgG2b; mast cell tryptase: IgG1kappa). The tissue sections were examined using an Olympus B× 40light microscope, and photographs were taken using a Nikon Eclipse80i light microscope using ImageJ software.

## Supplementary Material

Figure_Alt_text.docx

## Data Availability

The data that support the findings of this study are available from the corresponding author, JCB, upon reasonable request.

## ARRIVE guidelines

The contents of the manuscript adhere to the ARRIVE guidelines.
