# Supplementary material for: Expression of the cellular prion protein by mast cells in white-tailed deer carotid body, cervical lymph nodes and ganglia
Source: Prion. 2024 Sep 16;18(1):94–102. doi: 10.1080/19336896.2024.2402225 (PMC11409499; doi:10.1080/19336896.2024.2402225)
Supplement: Figure_Alt_text.docx [file KPRN_A_2402225_SM8635.docx]

ALT text for figures 1-5

1. Figure 1: Carotid bodies collected from white-tailed deer are easily identified using toluidine blue and hematoxylin stains in light micrographs due to their characteristic clusters of cells surrounded by a connective tissue capsule and many capillaries.

2. Figure 2: The 8H4 antibody shows the presence of the misfolded prion protein in

the obex of an infected white-tailed deer and the absence of the misfolded prion protein in the obex of an uninfected white-tailed deer.

3. Figure 3: Cells identified with an antibody specific to mast cells are similar in size,

shape, and distribution in white-tailed deer carotid bodies as cells that are identified using an antibody against the prion protein. When stains are combined on the same tissue sections the cells are identified as a single population; thus, mast cells express the prion protein in carotid bodies of white-tailed deer.

4. Figure 4: Mast cells express the prion protein in cervical lymph nodes, the

superior cervical ganglion, nodose ganglion and nerves located near the carotid bodies.

5. Figure 5: A number of structures associated with carotid bodies serve as potential routes for neuroinvasion of prions circulating in the blood, these include the superior cervical ganglion, nodose ganglion and cervical lymph nodes.
